# Momentary Affective States Are Associated with Momentary Volume, Prospective Trends, and Fluctuation of Daily Physical Activity

**DOI:** 10.3389/fpsyg.2016.00744

**Published:** 2016-05-23

**Authors:** Martina K. Kanning, Dominik Schoebi

**Affiliations:** ^1^Department of Sport Science, University of Konstanz, KonstanzGermany; ^2^Department of Psychology, University of Fribourg, FribourgSwitzerland

**Keywords:** exercise, emotion, ambulatory assessment, ecological momentary assessment, activity-trends, affect

## Abstract

Several interventions aiming to enhance physical activity in everyday life showed mixed effects. Affective constructs are thought to potentially support health behavior change. However, little is known about within-subject associations between momentary affect and subsequent physical activity in everyday life. This study analyzed the extent to which three dimensions of affective states (valence, calmness, and energetic arousal) were associated with different components of daily activity trajectories. Sixty-five undergraduates’ students (Age: *M* = 24.6; *SD* = 3.2; females: 57%) participated in this study. Physical activity was assessed objectively through accelerometers during 24 h. Affective states assessments were conducted randomly every 45 min using an e-diary with a six-item mood scale that was especially designed for ambulatory assessment. We conducted three-level multi-level analyses to investigate the extent to which momentary affect accounted for momentary volume, prospective trends, and stability vs. fluctuation of physical activity in everyday life. All three affect dimensions were significantly associated with momentary activity volumes and prospective trends over 45 min periods. Physical activity didn’t fluctuate freely, but featured significant autocorrelation across repeated measurements, suggesting some stability of physical activity across 5-min assessments. After adjusting for the autoregressive structure in physical activity assessments, only energetic arousal remained a significant predictor. Feeling energized and awake was associated with an increased momentary volume of activity and initially smaller but gradually growing decreases in subsequent activity within the subsequent 45 min. Although not related to trends in physical activity, higher valence predicted lower stability in physical activity across subsequent 45 min, suggesting more short-term fluctuations in daily activity the more participants reported positive affective valence. The current analyses afford interesting insight into within-subject associations between momentary affect and activity-trajectories in everyday life. Energetic arousal emerged as the only meaningful predictor of physical activity in daily life after adjusting for autoregression. A significant effect of valence on short-term activity fluctuations might indicate that activity interventions would benefit from taking into account enhancement of positive affective valence in everyday life. Moments of enhanced valence may scaffold attempts helping inactive people to get started with daily activities and overcome periods of inactivity in everyday life.

## Introduction

Strong evidence demonstrating health enhancing benefits of physical activity underscores the need for effective interventions to initiate physical activity behavior change (Physical Activity Guidelines Advisory Committee, 2008; Lee et al., 2012). Being physically active helps to prevent non-communicable diseases like coronary heart disease, type 2 diabetes, and some forms of cancer (World Health Organization, 2002; Wartburton et al., 2006). Despite its health relevance, most people don’t reach the recommended volume of being physically active for at least for 150 min per week (Troiano et al., 2008).

To increase physical activity, most interventions have been informed by social-cognitive theoretical models, such as theory of planned behavior (Fishbein and Ajzen, 1975), or the transtheoretical model (Prochaska and Velicer, 1997). Despite their well-established theoretical foundation, however, interventions showed mixed success in enhancing physical activity (Prestwich et al., 2014). Although these behavior change theories implicitly consider affective influences, behavior change interventions focus mainly on cognitive and reasoned determination and disregard affective influences. Yet, several studies have shown that affective constructs are relevant not only to explain variations in physical activity but also to predict prospective activities (for an overview: Ekkekakis et al., 2013). Incorporating affective influences in health behavior interventions may improve intervention effects (Helfer et al., 2015). So far, however, within-subject effects of affective states on subsequent physical activity are not well understood. The current study used an ambulatory assessment approach to examine whether and to what extent momentary affective states are associated with different components of daily activity-trajectories. Ambulatory assessments allow the repeated assessments of individual changes in physical activity and affective states over time in everyday life (Ebner-Priemer and Trull, 2009).

Prior studies pointed to the importance of affect in predicting health related behaviors. For instance, a questionnaire study (Lawton et al., 2009) assessed affective and cognitive attitudes of 390 healthy adults at time one to predict self-reported behaviors at a 1 month follow-up assessment. The study revealed that, compared to cognitive attitudes, affective attitudes were significantly more powerful predictors of alcohol use, smoking, exercising, and fruit and vegetable consumptions at time 2. Associations of affect with prospective physical activity were analyzed in a recent systematic review (Rhodes and Kates, 2015). The authors included 24 studies that measured affect in response to an acute or regular bout of physical activity, and that assessed enacted or intended physical activity as the dependent variable. Positive changes in affect during moderate physical activity was prospectively linked to more activity behaviors, whereas post-exercise affective responses were unrelated with activity. Williams et al. (2012) used a longitudinal design to examine whether affective valence during a treadmill walk of 10 min predicted physical activity. One hundred sixty four healthy, low-active adults participated in this physical activity promotion trail. Positive affective valence reported during the brief walk predicted concurrent physical activity and self-reported lifestyle activity 6 months later.

Ample evidence supports the importance of affect in predicting either physical activity intention to be active or the behavior of subsequent activity (Rhodes and Nigg, 2011). Based on this evidence, we argue that affect should be considered alongside cognitive and reasoned determination as factors explaining behavior change in physical activity (Ekkekakis et al., 2013). Several issues require clarification, however: first, most studies analyzed affective reactions due to a specific exercise program or treadmill walking and used these affect-perceptions to predict the intention to or the behavior of subsequent physical activity. Physical activities in daily living such as going for a walk, gardening, playing with children, or making a cycling tour have hardly received attention, although such lifestyle activities make up a large part of the entire volume of physical activities. Increasing this type of activity is important to prevent health related diseases. For instance, a prospective cohort study with more than 400.000 individuals (Wen et al., 2011) indicated that 15 min of activity a day was associated with a 14% reduction in all-cause mortality risk, and with a 3 years longer life expectancy. Second, the design of most former studies did not allow for analyzing associations of momentary affective states and physical activities as a process unfolding over time. Consequently, we know little about within-subject changes in daily physical activities following prior momentary affective states.

According to the Activity-State Hypothesis (Rowland, 1998) physical activity in everyday life seems to follow an homeostatic mechanism, indicating stability in dynamic systems by the process of negative feedback. That means that physical activity (or energy expenditure) might be overall stable over time and that after high volumes of activity low volumes are probably following and vice versa. Thus, physical activity in everyday life is highly predicted by former activity. This oscillating pattern of activity needs to be taken into account when analyzing variations in daily physical activities over time. If we assume a substantial concurrent association between affect and physical activity, activity is likely elevated at that time point and will likely decrease across subsequent measurements. As a result, potential positive prospective effects of affect on physical activity will be attenuated or masked; where potential negative prospective effects of affect on activity would be enhanced. To adjust for such bias when predicting subsequent physical activity from affect reports, it is important to take into consideration prior volumes of physical activity at any predicted measurement point of activity. This allows for predicting prospective physical activity variations independent of prior volumes of activity. Incorporating moment-to-moment (autoregressive) associations of physical activity reflects also a meaningful dimension of physical activity itself, namely its fluctuation or stability across time. For physical activity interventions, it is now relevant to know how important this autoregressive structure is in influencing activity compared to affective determinants.

**FIGURE 1:**
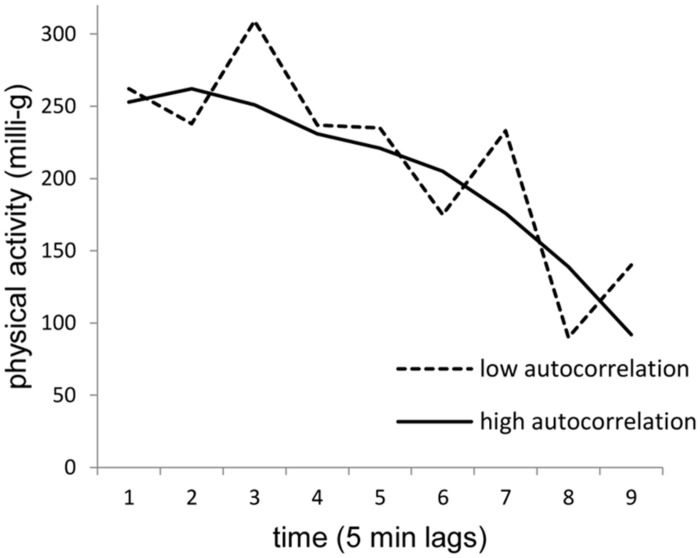
**Activity trajectories across time with low (broken line) and high (bold line) stability.** The graph exemplifies trajectories with a negative time trend after adjusting for autocorrelation. Moment-to-moment changes increase with time, on average, resulting in a curvilinear shape of trajectories.

The current study examined the extent to which momentary affective states predict momentary volume, prospective trends, and stability vs. fluctuation in daily physical activity. We used an ambulatory assessment approach to capture individuals’ momentary affective states and their physical activity in their daily lives. Based on the existing state of research (e.g., Kanning et al., 2013; Liao et al., 2015), we tested the hypotheses that valence and energetic arousal are positively whereas calmness is negatively associated with momentary volume of physical activity in daily life. We extended existing research in (1) analyzing to what extent the three different dimensions of affective states predicted prospective trends in physical activity, and (2) by investigating associations of momentary affect on stability vs. fluctuations of prospective physical activity across time. **Figure 1** provides examples for activity trends with high vs. low stability.

## Materials and Methods

### Subjects

A convenience sample of 65 undergraduate students (57% females) participated in this ambulatory assessment study. The sample was recruited from a German university and had a mean age of 24.6 years (*SD* = 3.2). The data were part of another study, which have been presented elsewhere (Kanning, 2013), but the analyses here and the combination of physical activity and affective states are unique to this paper.

Before data recording, subjects were informed about the aim of our study, which was analyzing the association between physical activity and affective states. All of the subjects took part in the study voluntarily. They received no monetary compensation for their participation. Following the process of institutional ethical approval, all of the participants provided written informed consent. APA ethical standards were followed in the conduct of the study.

### Ambulatory Assessment Procedure

Physical activity in daily living was measured continuously using a 3-way accelerometer (varioport-e; Becker Meditech, Germany) for 24 h. The varioport-e was started and then attached to each subjects’ hip. Twenty-four hours later, subjects returned to the laboratory where data recording was stopped. Participants were allowed to take off the accelerometer when sleeping. Ratings for affect were assessed via electronic diaries (Palm, Tungsten E2). For this, the palmtop prompted subjects every 45 min during a defined 14-h daytime period (8:00am–10:00pm). If the person made no entry to the electronic diary, the palmtop beeped again 10 min later to remind the person to fill in the questionnaire. The assessment always took place on weekdays. We included only reports that were made within 10 min of the first beep, which was the case for 87.9% of the reports. The average latency between the beep and the report was *M* = 2.15 min (*SD* = 3.96).

### Measures

#### Affective States

Momentary affective states were measured by using the Short Mood Scale (Wilhelm and Schoebi, 2007), which is based on the Multidimensional Mood Questionnaire, for which validity and reliability has been demonstrated (Steyer et al., 1997). The short scale is the only instrument that has been explicitly developed and evaluated for use in ambulatory assessment. The scale contains six items that assess the intensity of three dimensions of affective states that are ordered as semantic differentials: *valence* (unwell vs. well, discontent vs. content), *calmness* (relaxed vs. tense, calm vs. agitated), and *energetic arousal* (tired vs. awake, without energy vs. full of energy). Subjects answered the question “At this moment, I feel…” by moving a slider from the left end (e.g., unwell) to the right end (e.g., well) of the bipolar scale. Scores for each subscale were obtained by averaging the item scores, which resulted in a range from 0 to 5. Wilhelm and Schoebi (2007) investigated homogeneity at the between-person and the within-subject level. The reliability coefficient for the between-person level reached 0.92 for valence and 0.90 for energetic arousal and calmness. The reliability coefficient for the within-subject level reached 0.70 for valence and calmness and 0.77 for energetic arousal. Based on this finding, the reliabilities both resulted in good internal consistencies.

#### Physical Activity in Daily Living (PA)

The varioport-e measured acceleration (defined as change in velocity over time) and describes the intensity, the rate of occurrence and the duration of an actual physically active episode. Acceleration was measured in milli-g, for each minute in the 24-h period. All offline analyses and artifact-checks were performed by the interactive software package “Freiburg Monitoring System” according to a published procedure (Myrtek, 2004).

### Data Analysis

The data featured multiple dependencies, with activity measurements (level 1) being nested within affective state assessments at 45-min intervals (level 2), which were nested within participants (level 3). We therefore analyzed the data using multilevel models with three levels. To examine whether momentary affect predicted momentary activity volume, prospective trends, and fluctuation, we used a multilevel model in which we estimated a growth parameter for each 45-min interval for which participants reported about their affective states. To approximate a normal distribution, reduce short-term fluctuations, and reduce the amount of data, we aggregated activity scores for each 5 min interval, and then computed the moving average over series of three consecutive 5-min activity aggregates. To assess momentary volume of activity and prospective trends, and their associations with prior affect variables, we first ran a model that did not adjust for the autoregressive structure in daily physical activity (model 1). We then extended this model to incorporate an autoregressive structure (model 2). Comparing these models revealed to what extent associations between affect and different components of daily activity-trajectories were accounted by prior activity volumes.

In model 1, we tested a growth model at level 1, with the nine moving average activity scores (45/5 = 9) changing as a function of time, for each 45-min interval between affect measurements. Minor deviations from 45-min were possible due to response latency. This model allowed for momentary activity volume and prospective trends to vary within person across 45-min lags. For example, activity could increase over the 45 min, and then decrease across the subsequent 45 min, before remaining stable across the third subsequent 45-min interval. The level-1 equation for model 1 can be written as:

$${Activity}_{i+1jk}=b_{0}\,{(cons\,\tan t}_{ij})\,+b_{1}\,{(time}_{ijk})\,+e_{ijk}$$

**FIGURE 2:**
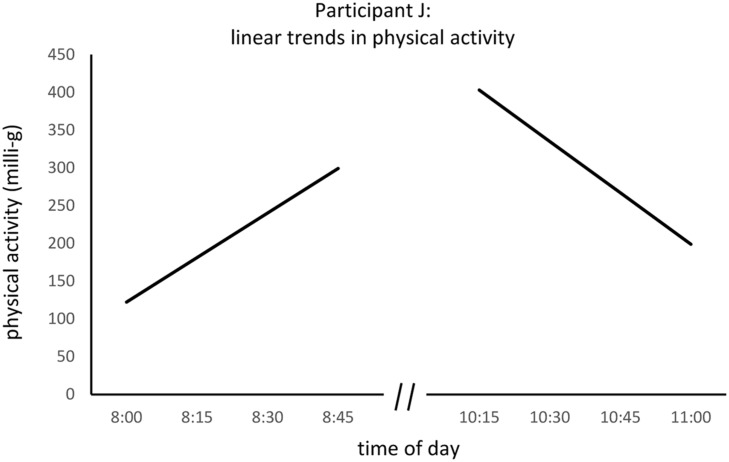
**Examples of linear activity trends across time for two 45-min periods of participant J.** The graphs exemplify 45-min trends in activity, reflecting increases (left slope) and decreases (right slope) in the activity of the same participant on the same day.

The activity following one time unit (5-min) after a particular time point *i*, during the 45-min lag *j* of person *k*, is represented by a constant *b*_0_, capturing the momentary activity volume at the beginning of the 45-min lag, a time parameter *b*_1_, reflecting trends in activity across the 45-min time lag, and an error term *e*_ijk_. A positive time trend reflects increasing activity across a particular 45-min period, and a negative time trend reflects decreasing activity across a 45-min period, as exemplified in **Figure 2**. We also examined extended growth models, including squared and cubic time trends to capture curvilinear associations. Because these models did not significantly improve the predictions, we report only the linear growth models.

At level 2, we then used the affect reports at each 45-min interval to explain variability in (a) momentary activity volume, and (b) trends of activity across 45 min intervals.

Level-2 equations (model 2):

$$b_{0}\,\;=\;g_{00}\,+\;g_{01}\,\;{(affect}_{jk})\,+\;u_{00}$$

$$b_{1}\,\;=\;g_{10}\,+\;g_{11}\,\;{(affect}_{jk})\,+\;u_{01}$$

Parameter *g*_01_ reflects the extent to which momentary activity volume at the beginning of the 45-min lag j of person *k* is a function of that person’s affect report at the beginning of lag j. The estimate for *g*_11_ captures the association of a person’s affect report at the beginning of lag j, and his or her trend in activity across that lag. Finally, *u*_00_ and *u*_01_ represent residual variance of the constant and the linear growth parameter.

In model 2, we added the first order autoregressive parameter to capture the stability of activity across time. This parameter captures moment-to-moment stability (or instability), and adjusts the time trajectories for regression toward the mean by eliminating the effects of prior volumes of activity. Thus, in this model, the time parameter no longer captures constant linear trends of activity across 45-min intervals, but the extent to which activity changes between 5-min activity measurements become increasingly positive or negative over the 45-min intervals. The full level-1 equation (model 2) can be written as follows:

$${Activity}_{i+1jk}\,=\;b_{0}\,\;{(cons\,\tan t}_{ij})\,+\;b_{1}\,\;{(time}_{ijk})\,+\;b_{2}\,\;{(activity}_{ijk})\,+\;e_{ijk}$$

In addition to the equation for model 1, the equation now includes an autoregressive parameter *b*_2_, capturing stability in activity across time.

In addition to regressing momentary activity volume (*g*_01_) and trends of activity across 45 min intervals (*g*_11_) on affect reports, we also estimated whether stability or fluctuation in activity across time was predicted by affect (*g*_21_).

Level-2 equations (model 2):

$$b_{0}\,=\;g_{00}\,+\;g_{01}\,\;{(affect}_{jk})\,+\;u_{00}$$

$$b_{1}\,=\;g_{10}\,+\;g_{11}\,\;{(affect}_{jk})\,+\;u_{01}$$

$$b_{2}\,=\;g_{20}\,+\;g_{21}\,\;{(affect}_{jk})\,+\;u_{02}$$

The estimate for *g*_21_ captures the association of a person’s affect report and stability in her activity across repeated measurements, and *u*_02_ represents the residual variance of the autoregressive parameter across 45-min lags.

Importantly, the affect predictors were centered at each individual’s mean affect across all measurements, and therefore, the estimates for these parameters reflected within-subject associations between affect and activity volumes, and within-subject stability. We ran several models in which only one (as shown in the level-2 equations above) or multiple affect components (valence, energetic arousal, and calmness) were tested as predictors of momentary activity volume, trends, and stability estimates. In the model with all three affect components, no significant effects emerged that were not significant in the models testing each component separately, and we therefore report the results from the combined test.

## Results

### Descriptive Statistics

The average activity level across individuals was *M* = 90.25 milli-g (between-subject *SD* = 27.31 milli-g), while the median was *Md* = 54.99 milli-g (between-subject *SD* = 20.31 milli-g). For comparison purposes, jogging episodes produce approximately 1,000 milli-g/min, walking episodes produce approximately 350 milli-g/min, and sheer sitting episodes produce approximately 10 milli-g/min (Kanning et al., 2013). The results for testing level-1 predictors largely confirmed our expectations. Estimating linear trajectories across 45-min periods (model 1) indicated no significant overall trend (*b* = –0.163, *t* = 0.565, *p* = 0.574). The average activity change between repeated measures was 0.14 (*SD* = 0.82) and did not differ significantly from zero (95% CI = –0.07; 0.35), thus suggesting that activity fluctuated around a habitual level. Examining first order autoregression suggested significant stability in activity across time (*b* = 0.718, *t* = 40.760, *p* < 0.001). The average level of valence was *M* = 3.56 (between-subject *SD* = 0.60), of energetic arousal *M* = 3.02 (between-subject *SD* = 0.56), and calmness *M* = 3.43 (between-subject *SD* = 0.60), indicating moderate to high values of momentary affect with substantial within-subject variation (cf. **Table 1**).

**Table 1:** Descriptive statistics of the three dimensions of affective states and physical activity.

Variable	Between-subject		Within-subject variability		Minimum	Maximum
	Mean	*SD*	Mean	*SD*		
Physical activity	90.25	27.31	104.94	47.42	0	1330.71
Valence	3.56	0.6	0.85	0.31	0	5
Energetic arousal	3.02	0.56	1.16	0.29	0	5
Calmness	3.43	0.6	0.89	0.33	0	5

*Within-subject variability is indicated as standard deviations.*

### Did Momentary Affect Predict Momentary Activity Volume, Prospective Trends, and Stability vs. Fluctuation in Daily Physical Activity?

Momentary activity volume, prospective trends, and stability varied significantly across 45-min intervals (*p*s < 0.001), but autocorrelation and time trends did not vary across individuals (*p*s > 0.421).The results for the level-2 for model 1 and model 2 are summarized in **Table 2**.

**Table 2:** Within-subject relations between the three dimensions of affective states (valence, calmness, and energetic arousal) and different components of daily activity-trajectories (momentary volume, trend, and stability).

Level 2 parameter	Momentary activity volume (intercept)			Trend (time slope)			Stability/fluctuation (activity slope)		
	Coefficient	*t*-ratio	*p*-value	Coefficient	*t*-ratio	*p*-value	Coefficient	*t*-ratio	*p*-value
**Model 1**						
Valence	11.797	2.827	0.005	-1.486	2.466	0.014			
Calmness	-12.176	3.264	0.001	1.031	1.920	0.055			
Energetic arousal	20.521	7.502	<0.001	-0.779	1.974	0.049			
**Model 2**						
Valence	6.955	1.417	0.157	-0.489	1.297	0.195	-0.031	2.224	0.026
Calmness	-7.992	1.814	0.070	0.206	0.613	0.540	0.012	0.904	0.366
Energetic arousal	19.892	6.174	<0.001	-0.610	2.474	0.013	-0.008	0.793	0.428

Estimates for model 1 suggested that at times reports of more positive affective valence (*b* = 11.797, *p* = 0.005) less calmness (*b* = –12.176, *p* = 0.001), and more energetic arousal (*b* = 20.521, *p* < 0.001), were associated with higher momentary activity. Affective states also predicted activity trends across time. Reports of more positive affective valence (*b* = –1.486, *p* = 0.014), and more energetic arousal (*b* = –0.779, *p* = 0.049) were associated with activity decreases over the subsequent 45-min intervals. A marginally significant parameter suggested that reports of more calmness were associated with activity increases (*b* = 1.031, *p* = 0.055). Importantly, the pattern of estimates for prospective trends was complementary with that of activity volumes, suggesting a trend toward mean activity after activity peaks and low points.

When taking into account prior activity volumes in model 2, a significant association between affective states and activity trends resulted only for energetic arousal. In cases when participants reported more energetic arousal, activity changes trended toward decreases, whereby the decrease was initially small but gradually growing across the subsequent 45-min interval (*b* = –0.610, *p* = 0.013).

Positive affective valence was associated with less positive (more negative) autoregressive parameter estimates (*b* = –0.031, *p* = 0.026), suggesting that within 45-min intervals after participants reported better feelings, physical activity was less stable, or in other words, fluctuated more strongly across 5-min measurements.

## Discussion

This ambulatory assessment study was among the first to investigate different components of daily activity-trajectories (momentary volume, prospective trends, and stability vs. fluctuation) and to analyze to what extent affective states predicted trends and stability independent from prior volumes of activity. The results suggested that momentary affective states were significantly associated with momentary volume of physical activity. Furthermore, energetic arousal is negatively associated with prospective activity trends, whereas valence is positively associated with prospective fluctuations of physical activity.

To our knowledge, trajectories in daily physical activity have hardly been investigated in detail. So far, several studies investigated patterns of physical activity, analyzing how much time the sample was sedentary or physically active with moderate or high intensity (e.g., Evenson et al., 2015; Hooker et al., 2015). Although these studies described the quantity of different activity volumes, they did not analyze trajectories in physical activity. The findings of the presented study suggested that the volume of physical activity in daily life varies over time without significant short-term trends. Thus, physical activity in everyday life oscillate around a habitual level, supporting the Activity-State Hypothesis (Rowland, 1998). The Activity-State Hypothesis has been predominantly used to explain compensatory change in one domain (e.g., leisure-time), when physical activity is being increased or decreased in another domain (e.g., school, at work; for an overview Gomersall et al., 2013). Although the presented study investigated daily physical activities without distinguishing between different domains, the findings suggested that physical activity might follow an autoregressive structure. To create effective interventions for enhancing physical activity, it is important to understand whether and to what extent affective states can influence daily physical activity in addition to the autoregressive structure of activity.

Valence, energetic arousal, and calmness were all significantly associated with the momentary volume of daily life activity. These findings converge with former ambulatory assessment studies on associations between physical activity and affective states in everyday life. However, most of these former studies tested the effects of physical activities on affective states, rather than vice versa (for an overview: Kanning et al., 2013). Nevertheless, a recent systematic literature review examined six studies that tested whether affective states predicted subsequent physical activity (Liao et al., 2015). For instance, in a 12 h- ambulatory assessment with 124 healthy adults between 18 and 73 years, Schwerdtfeger et al. (2010) assessed physical activity continuously and affective states every hour during waking hours. The authors examined associations between momentary affect and the average activity volumes during four subsequent periods (1, 1–5, 1–15, and 1–30 min). Their results suggested that the more positive subjects felt, the more active they were during all four subsequent activity-periods. However, the other studies included in Liao’s et al. (2015) review did not consistently report positive associations, suggesting overall mixed support for the assumption that positive feelings lead to physical activity.

The focus of the current research was on different components of daily activity-trajectories and whether these activity variations were predicted by affective states in daily life. The findings of model 1 suggested that patterns of change in momentary activity volumes corresponded with prospective activity trends. Feeling well (valence), full of energy (energetic arousal) and agitated [calmness (–)] were each associated with a high level of momentary volume of the physical activity aggregated across the 5 min directly following the e-diary assessment and with decreases in activity during the subsequent 45 min. These results suggested a trend toward mean activity after activity peaks. To address that autoregressive structure and because high activity would naturally regress to the mean and therefore spuriously inflate negative associations between affect dimensions and activity, we integrated an autoregressive parameter in model 2, which allowed us to predict activity-trends independently from prior volumes of activity. This model 2 revealed that calmness and valence were no longer meaningful predictors of activity changes. However, energetic arousal remained a significant predictor for momentary volume and prospective trends of daily physical activity. Thus, when an individual felt highly energized, his or her activity volume was high and decrease across the subsequent 45 min interval. Interestingly, decreases in activity were initially small but gradually growing across the subsequent 45-min interval. So first, participants hold their activity volume followed by an increasing decrease in subsequent physical activity (cf. **Figure 1**).

Beside the function to control for the presumed biological mechanism, the autoregressive parameter itself captures stability of physical activity across time. The significant and negative association of valence pointed to more fluctuations in activity the better the person felt before. Positive affective valence appears to foster changes in activity volumes in everyday life. According to Fredrickson’s *broaden and build model* positive emotions (e.g., joy, contentment, and love) broaden individual’s repertoire for action to seek new goals, for instance, and expend individual’s resources and friendships (Fredrickson, 1998). Thus, individuals are ideally situated to think and act in ways that promote both resource building and involvement with approach goals. Although this model is about the effect of general positive emotions and does not reflect on potential effects of momentary affect in everyday life, it may hint at how positive affective valence may operate in daily life. It may be that positive feelings support people more readily engaged in doing the things that they want or have to do in their everyday life (e.g., run some errands, going for a walk), and therefore, lead to more frequent changes of the specific types of activity. In most cases, such engagement is associated with an increased variety of physical activity. The effect of positive affective valence on prospective physical activity may also be interesting for physical activity interventions. It is a challenge for inactive people to bring themselves to an active lifestyle. If positive feelings help individuals to get started with daily activities, it may be easier for them to overcome periods of inactivity in everyday life. Thus, interventions to increase daily activities may be more effective if they provide strategies for emotion regulation in addition to prescriptions how to be active in daily life. If an inactive person is able to experience positive feelings, it will be more likely that this person engages in the recommended activity. To enhance positive feelings in everyday life, an intervention may support inactive individuals in selecting and modifying everyday life situations, which are individually associated with positive feelings and which may be suitable to be physically active.

Some limitations need to be taken into account when interpreting the results. First, physical activity and affective states were assessed only during the course of a single day. To better support the existence of a biological mechanism and to analyze trajectories in daily physical activity, the assessment period needs to cover more than 1 day. Second, this study involved only university students, which resulted in a sample that is both younger and of a higher socioeconomic status than the overall population. Generalizations based on these results thus need to be done with caution. Lastly, this study did not assess important environmental (e.g., weather, physical environment) or psychosocial (e.g., being together with meaningful people, social support) factors, which might have influenced both momentary affect and physical activity.

## Conclusion

Broad evidence indicates that affect is an important predictor of physical activity motivation and behavior. Physical activity interventions should therefore make use of affect and related constructs to improve their effectiveness to induce behavior change. Our results suggest that important dimensions of momentary affect – valence, calmness, and energetic arousal – were all significantly associated with momentary volume and prospective trends in physical activity in daily life. After adjusting for the autoregressive structure, and thereby adjusting for prior activity levels when predicting prospective physical activity, calmness and valence were no longer significantly related to prospective activity trends, whereas energetic arousal remained a significant predictor. Energetic arousal thus emerged as the only meaningful predictor of momentary activity volumes, and of prospective trends in activity changes. Specifically, feeling highly energized predicted initially smaller changes that grow larger over the span of the 45 min periods. In addition, positive affective valence was predictive of short-term activity dynamics, as reflected by a significant association with more short-term fluctuations (or less stability) in everyday life activity volumes. These findings could imply that interventions designed to enhance daily physical activity might be more effective if they incorporated strategies to foster positive affective valence in everyday life. Strategies to enhance positive feelings may help inactive people to initiate change their inactive lifestyles.

## Author Contributions

MK made substantial contributions to the design of the work and conducted the study. MK interpreted the data, drafted the work, and gave the final approval of the version to be published. DS made substantial contributions to the analysis and the interpretation of the data. DS revised the work critically for important intellectual content and gave final approval of the version to be published. MK and DS both agreed to be accountable for all aspects of the work ensuring that questions related to the accuracy or integrity of any part of the work are appropriately investigated and resolved.

## Conflict of Interest Statement

The authors declare that the research was conducted in the absence of any commercial or financial relationships that could be construed as a potential conflict of interest.
